# Muscle Activation Profiles of Lower Extremities in Different Throwing Techniques and in Jumping Performance in Elite and Novice Greek Judo Athletes

**DOI:** 10.2478/hukin-2013-0026

**Published:** 2013-07-05

**Authors:** Georgios Zaggelidis, Savvas Lazaridis

**Affiliations:** 1 Department of Physical Education and Sports Science, Aristotle University of Thessaloniki, Greece.

**Keywords:** electromyography, judo, vertical jumping, hip throw, co-contraction ratio

## Abstract

The aim of this study was to examine the neuromuscular adaptations of knee muscles during hip throwing techniques and vertical jumps in elite and novice Greek judokas. Ten elite and ten novice judokas performed two hip throws and different vertical jumping tasks. Surface electromyograms were recorded from vastus lateralis and biceps femoris muscles along with generated kinetics. Elite judokas revealed higher EMG activity of agonist muscles during throws and jumps but lower co-activation levels. Better jumping performance, better utilisation of the stretch-shortening cycle mechanism, higher and earlier generated push-off forces and shorter contact time periods characterized elite judokas. Total neuromuscular activation that adopt elite judokas reveals a more mature and skill dependent strategy compared to novice ones.

## Introduction

Competitive judo is described as a multi-joint and high intensity movement discipline, in which specific throwing techniques require good physical fitness ( Franchini et al., 2003 ). Judo is a martial art classified among those specialties requiring high technical skills along with execution of main technical actions as fast as possible ( Franchini et al., 2007 ; Thomas et al., 1989 ). Neuromuscular activity during specific ballistic movements of martial sports has mostly been studied on karate movements ( Sforza et al., 2002 ; Sorensen et al., 1996 ; Zehr et al., 1997 ). To date, several studies have investigated judo from a biomechanical perspective, but only few included electromyography in order to further examine the underlying mechanisms and adaptations in specific events such as throws in judo ( Franchini et al., 2011 ; Franchini et al., 2013 ; Pucsok et al., 2001 ). In addition to this, it would be interesting to study secondary ballistic and explosive movements such as vertical jumping tasks from a neuromuscular point of view and to give information about the potential different neuromuscular activity patterns adopted as a result of different training level.

For these reasons, the main target of this study was to identify the neuromuscular differences during specific judo throwing techniques as a consequence of years of specific training in elite and novice Greek judo athletes. Secondly, EMG and kinetic differences of lower extremities in selected maximal vertical jumping tasks were examined to further support the factor of neuromuscular control responses as a result of training status.

Potential neuromuscular adaptations due to different training background would explain the differences in performance between the two examined groups. This would be obvious by examing the electromyography patterns in the lower extremity muscles during the execution of real-time movements occurring during a simulated fight as well as during fundamental vertical jumping tasks which determine the sports level of an athlete during fast-explosive movements.

## Material and Methods

### Participants:

The two groups of participants were divided according to their category and due to their sports level (novice/elite) as well as sex [10 elite (8 males/2 females) and 10 novice (8 males/2 females)]. All participants had at least 5 years of national competition experience and the elite group had international experience. Data including age, body mass, and body height were collected for all participants and are presented in Table 1 .

### Measures:

Kinetic data were recorded with a ground mounted 40 x 60 cm force plate (Bertec Type 4060, Bertec Corporation, Columbus, OH, USA). The sampling frequency for ground reaction force signals was set at 100 Hz. The EMG activity was recorded with a BTS Telemg EMG device (Milano, Italy), using bipolar surface Ag/AgCl electrodes (contact surface 0.8 cm, inter-electrode spacing 2 cm). The signal was preamplified (1,000x). The sampling frequency for the EMG was set at 1 kHz.

### Procedures:

All participants signed informed consent, consistent with Aristotle University guidelines. Initially, the investigator who assessed all evaluations measured the anthropometric characteristics. Then participants warmed up on a cycle ergometer and performed a specific judo warm-up. EMG was measured for the dominant limb only, as determined by the preference limb to kick a ball intuitively ( Wikstrom et al., 2006 ). The EMG electrodes were placed following the SENIAM guidelines ( Hermens et al., 2000 ) over the belly of the vastus lateralis (VL) and the long head of the biceps femoris (BF). The ground electrode was placed over the bony surface of the contra lateral wrist. Skin was shaved and cleaned with an alcohol solution and skin impedance was maintained below 2 kΩ. EMG signals were filtered with a zero lag, second order, band-pass (10–500 Hz), Butterworth filter, followed by full-wave rectification and then low-pass filtering at 50 Hz. The measurements were recorded in a well-simulated and controlled environment. The subject was instructed to perform two throwing conventional hip techniques [Harai-Goshi-Hip Sweep Throw (HG), and Uchi Mata-Inner Thigh Throw (UM)] with an adequate combination of maximal effort and proper technique and in random order each time. This was repeated three times for each technique with a rest of 30 s between throws. The conventional hip techniques in Judo, the Uke (receiver) is performed in a basic natural standing position with feet wide apart and body bent slightly forward. Thus, the subject performed a throw with maximal effort while maintaining his balance (staying the support foot on the force plate). This procedure was designed to simulate the throw under ideal conditions with the three general phases of all judo throwing techniques. For the purpose of our study regarding EMG activation, the first two phases were analysed, where the preparatory phase defined as breaking an opponent’s balance and the process of fitting into the throw were recorded. Each participant during the throwing conditions, served both for the Tori (thrower, the one who throws) and the Uke (receiver, the one who receives the throw) and only with the participant who competed.

After the execution of the specific-judo movements, the jumping measurements followed. The jumping test included three maximal efforts at each jumping condition with randomized order and with 2-min rest interval in between. For the squat jump (SJ), subjects were positioned on the force plate with the knee angle set at 90º. The starting knee angle was recorded using a standard goniometer. From this position, the participants were asked to jump as high as possible into an upright position. For the countermovement jump test (CMJ), subjects stood erect, and counter-moved until the knee was flexed approximately to 90º, before jumping. The same instructions for jumping were given. Finally, regarding the drop jump from 20cm (DJ20), subjects landed on both feet on the force platform, which was placed approximately 8 cm in front of the jumping platform edge and following this, they performed a maximum vertical jump. For all types of jumps, hands were placed on the hips and subjects were instructed to jump as high as possible. During the test no verbal feedback about the performance was provided. Three trials with maximal effort were captured with forefoot landing, as judged from kinematic data and with the typical shape of force–time curve, as described previously ( Kovacs et al., 1999 ).

### Analysis:

Data were further processed online using scripts of Matlab 6.1 (The MathWorks Inc.). All trials were averaged. Jumping height was estimated taking into account the impulse which was recorded from the vertical ground reaction force (vGRF)–time curve. Only the best trial was further analyzed. The peak vGRF was normalized to body mass of each subject. The timing of peak value in VGRFs in milliseconds (ms) was calculated from the force-time curve ( Lazaridis et al., 2010 ). The time of contact with the ground was derived from the force plate after the vGRF exceeded 20 N. Electromyographic parameters were calculated for different phases. Firstly, regarding hip throwing techniques, the timing period which was analysed was only that when throwers’ support leg was onto the platform and particularly from the moment when the opponent’s balance disturbed till thrower toed-off the platform. For the jumping tasks, firstly for the SJ, the propulsion phase was analysed, starting from movement initiation until the take-off. For the CMJ and DJ20 tests, the braking and the propulsion phase were determined by the ground reaction force-time curve. The mean muscle activation was calculated for each phase of the jump. The EMG signals were normalized to those of SJ, which was considered as the maximum value ( Liu et al., 2006 ). Lastly, the co activation of the BF was expressed as the ratio of BF/VL at each sub phase of jumping tasks and in the main phase of hip throws.

Statistics were performed with the SPSS/PC 16.0 (SPSS Inc.) statistical package. Mean and standard deviation for all dependent variables were assessed. An analysis of variance (ANOVA) was performed to test, when possible, the effect of group (elite judokas and novice ones) on the vertical jump task (SJ, CMJ and DJ20) and its all EMG and kinetic parameters of each task, and on EMG activation and cocontraction in the two hip throwing techniques in both groups. The Mann-Whitney non-parametric test was used to identify significant differences between the groups. The significance level was set at 0.05 for all tests performed.

## Results

The absolute values of VGRFz were higher in all examined techniques in elite judokas compared to novice ones. The same was true when these values were normalized to each participant’s body weight (BW). In all throwing techniques and particularly in the UM technique, elite judokas generated higher relative VGRFs compared to novice ones. Regarding the timing of appearance of this peak VGRFs, in all examined throwing techniques elite judokas presented earlier generation of this magnitude (p=0.005). In fact, elite participants applied these VGRFs almost 110 to 140 ms following the onset of each throwing technique. The same did not occur in case of novice judokas who presented their peak values of VGRFs almost 200ms after the same onset. As for the EMG activation during throws, both agonist and antagonist activation of the support leg was recorded during the two types of throws. Elite judokas in both types presented higher EMG values of the agonist muscle (VL), but lower values of antagonist muscle (BF) in the same phase compared to their novice counterparts.

**Figure 1:**
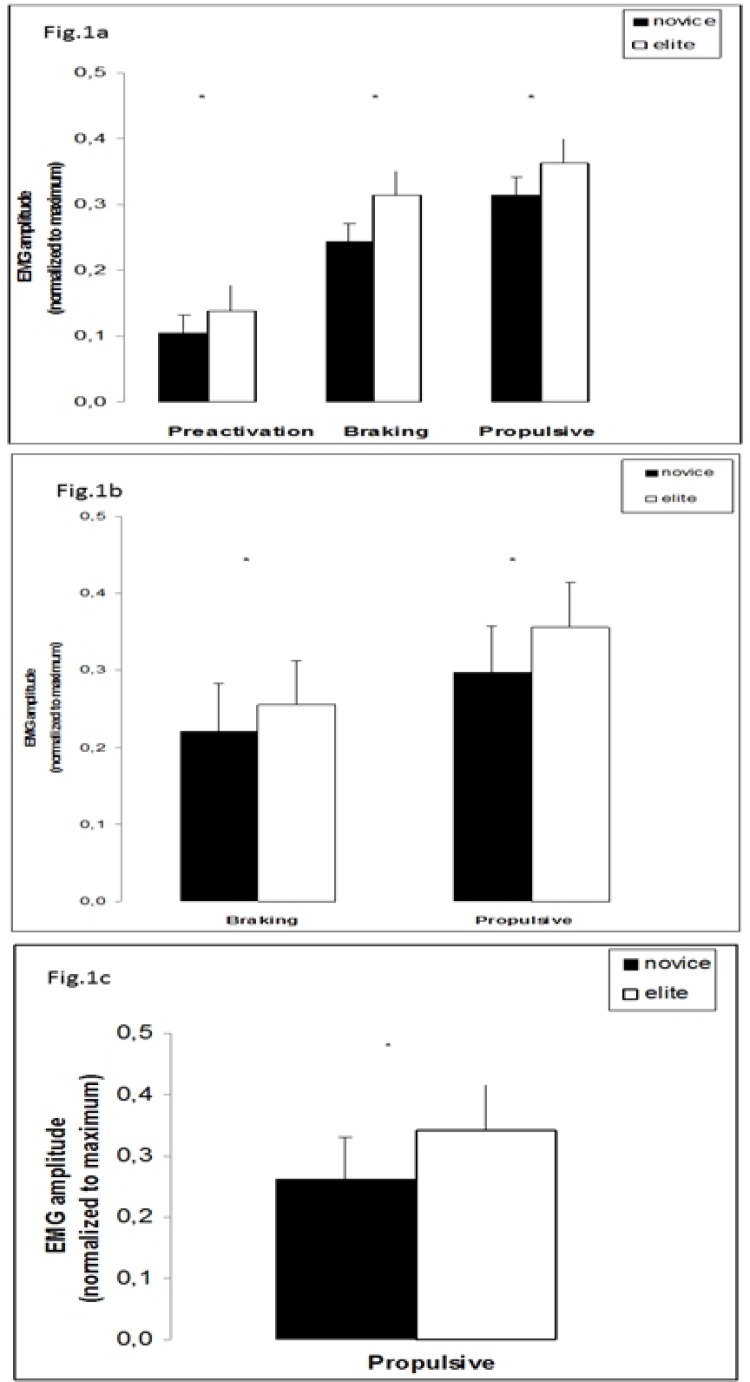
*
VL EMG amplitude during jumping tasks (a:DJ20, b:CMJ and c:SJ) in all examined phases in novice and elite judo athletes. Asterisks indicate statistical significance between the groups.
*

**Figure 2:**
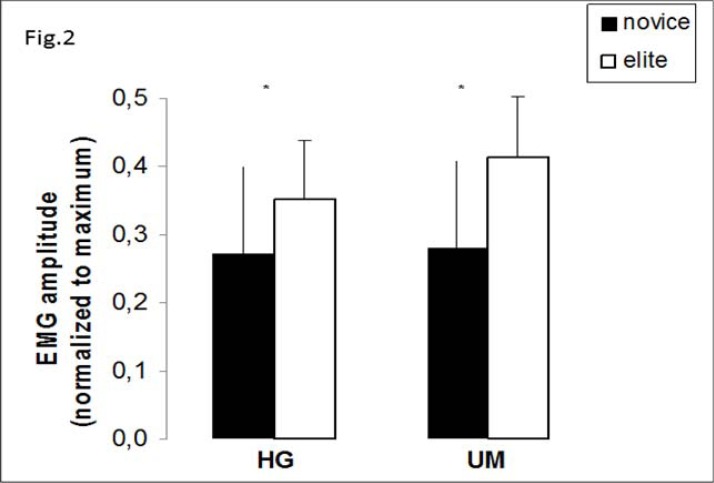
*
VL EMG amplitude during the preparatory phase of Harai-Goshi-Hip Sweep Throw (HG), and Uchi Mata-Inner Thigh Throw (UM) in novice and elite judokas. Asterisks indicate statistical significance between the groups.
*

Regarding jumping tasks, elite judokas presented better jumping performance in all examined tasks (SJ, CMJ and DJ20) compared to novice ones. Particularly, the better performance was observed during the CMJ task and this occurred as a result of a series of kinetic parameters and adaptations which can be described as follows: elite judokas presented shorter contact times, greater and faster absolute and relative vGRFs ( Table 2 ). These results along with higher EMG values of agonist muscle in all sub phases of jumps, led to a better utilisation of the stretch shortening cycle, which was visible in the CMJ. The antagonist activation of BF muscle was at lower levels in elite judokas compared to novice ones in examined phases of SJ, CMJ and DJ20. All the above mentioned results are presented in Tables 2 and 3 and Figures 1 and 2 .

## Discussion

This study was conducted to characterize from a neuromechanical point of view the jumping performance in three different vertical jumping tasks and in two popular hip throwing techniques of elite and novice Greek judokas. The major findings obtained in the present study were:

1. During the selected jumping tasks, elite judokas presented better jumping performance, as well as recoil utilization of the SSC, greater production of relative vGRFs in the push-off phases and shorter contact times at each examined phase. All the above, in fact characterized the parameters which favor better jumping performance as it was observed.
2. The neuromuscular activity pattern obtained during the selected jumps revealed important differences between groups. Elite judokas presented higher VL EMG activity (amplitude) in preactivation, eccentric and propulsive phase of all jumps (where preactivation and eccentric phase examined). In addition to this, co-activation (BF/VL ratio) in the same tasks at each sub phase of jumping task was significantly higher in the novice group. Novice participants presented higher levels of antagonist activity with respect to elite ones in all jumps.
3. The neuromuscular activity pattern during the execution of the two different hip throwing techniques revealed significant differences between the two groups. Higher values of EMG activity, earlier and higher generated vGRFs in the support leg were prominent characteristics in the group of elite judokas. Reduced antagonist muscle activation was also presented in the support leg in the same group (elite) in both throwing techniques.

A recent study ( Zaggelidis et al., 2012 ), which examined the vertical jumping performance among advanced judo athletes and untrained ones concluded that this performance is closely related to the training background. In fact, these athletes revealed superior ballistic performance and better neuromuscular adaptations as a result of their training. Similarly, in our study, elite judokas showed superior ballistic performance, indicated by greater relative peak force and faster generated forces in all examined tasks (jumps, throws).

This finding comes in accordance with a previous study that referred to combat sports and supports the neural and muscular adaptations due to their training ( Amiridis et al., 1996 ). In this study, it was concluded that three possible mechanisms might explain this differentiation in ballistic performance, mechanisms that the current study has already revealed and are true in case of elite judokas. Increased agonist activation, reduced antagonist activity and higher relative peak force production are common characteristics with the above mentioned study which reinforce our results. Reduced antagonist activity in a specific task is a pattern arising from the habitual skill motor acquisition ( Bernardi et al., 1996 ; Croce et al., 2004 ). Elite judokas, both in simple tasks (SJ) and more complex (CMJ, DJ20 and throws) manage to adopt a simple EMG activation pattern which is characterized by agonist muscle activation in high levels and minimal intervention of antagonist activation. The same EMG profile was also adopted in a recent study by a group of elite karate athletes who as a result of their training background presented a more efficient activation strategy with all the above mentioned EMG characteristics compared to novice karatekas ( Sbriccoli et al., 2010 ). Participation and years of adaptation to ballistic and explosive strength training and particularly in judo specific training may be the major factor which induced specific neuromuscular alterations as previous studies in martial arts suggested.

In conclusion, elite judokas adopt a different neuromuscular activation pattern compared to novices, which is mainly attributed to their different skill and training background and this fact is obvious on a electromyographic and kinetic level. Elite judokas presented higher EMG agonist but lower antagonist activation in all selected tasks, higher and faster generated relative vertical ground reaction forces during support phases of tasks and in general better utilisation of stretch-shortening cycle mechanism due to their specific judo training adaptations. Future studies, using both electromyography and kinematic analysis during simple and complex tasks in the same groups, will give accurate conclusions to the theme of neuromuscular alterations and adaptations as a result of specific training.

## Figures and Tables

**Table 1:** *
Participants’ physical characteristics (Values expressed as mean ± standard deviation)
*

	** Novice (n=10) **	** Elite (n=10) **
Sex (male/female)	8/2	8/2
Age (y)	21,5±4,6	20,2±4,3
Body height (cm)	175,6±5,9	176,5± 5,4
Body mass (kg)	87,7±21	86,8± 16,1
Body fat (%)	20,2±2,7	15,6± 3,5

**Table 2:** *
Kinetic data during the two examined techniques and the vertical jumping tasks in novice and elite participants (Values expressed as mean ± standard deviation)
*

	** Novice (n=10) **			** Elite (n=10) **		
** Type of throwing **	** Harai-goshi **	** Uchi mata **		** Haraigoshi **	** Uchi mata **	
Peak Fz (N)	2892±606	2964±694		3027±493 *	3278±518 ***	
Normalized Fz to BW	3,3±0,2	3,4±0,5		3,5±0,4 *	3,9±0,8 *	
Timing of Peak Fz (ms)	178±29	197±27		117±33 **	127±20 **	
** Jumping Task **	** SJ **	** CMJ **	** DJ20 **	** SJ **	** CMJ **	** DJ20 **
Jump height	22,2±4,1	24,8±3,8	22,2±2,7	26,6±4,3 *	30,6±3,3 **	27,4±6,2 *
Contact time (ms)	292±85	375±95	382±102	221±67 *	312±93 *	348±133 *
Gain of Stretch Shortening Cycle * [(CMJ-SJ)/CMJ] * (%)		10,4± 3,4			13± 4,5	
Peak vertical ground reaction force during push-off phase (Newton/Body mass)	2,3±0,4	2,9±0,5	3,5±0,4	2,8±0,5 *	3,2±0,4 *	4,2±0,5 * *
Time to Peak Fz during push-off (ms)	152±39	141±42	129±26	123±44	111±35 *	101±42 *

*, **, ***: *significant difference between the two groups (p<0.05, p<0.01, and p<0.001, respectively)*

**Table 3:** Co-activation (BF/VL ratio, in mV) during the two examined techniques and the vertical jumping tasks in novice and elite participants (Values express mean ± standard deviation)

	** Novice (n=10) **			** Elite (n=10) **		
** Type of throwing **	Harai-goshi	Uchi mata		Haraigoshi	Uchi mata	
EMG amplitude (mV)	0,72±0,1 ***	0,66±0,1 *		0,43±0,1	0,39±0,1	
** Jumping Task **	** SJ **	** CMJ **	** DJ20 **	** SJ **	** CMJ **	** DJ20 **
Braking phase (mV)	-	0,68±0,2 **	0,73±0,1 **	-	0,38±0,1	0,37±0,1
Push-off phase (mV)	0,76±0,1 **	0,69±0,2 *	0,67±0,2 *	0,43±0,1	0,5±0,1	0,42±0,1

*, **, ***: *significant difference between the two groups (p<0.05, p<0.01, and p<0.001, respectively)*
